# Supplementary figures and images for: A family of interaction-adjusted indices of community similarity
Source: ISME J. 2016 Dec 9;11(3):791–807. doi: 10.1038/ismej.2016.139 (PMC5322292; doi:10.1038/ismej.2016.139)

SparCC Correlations ( $I_C$ )

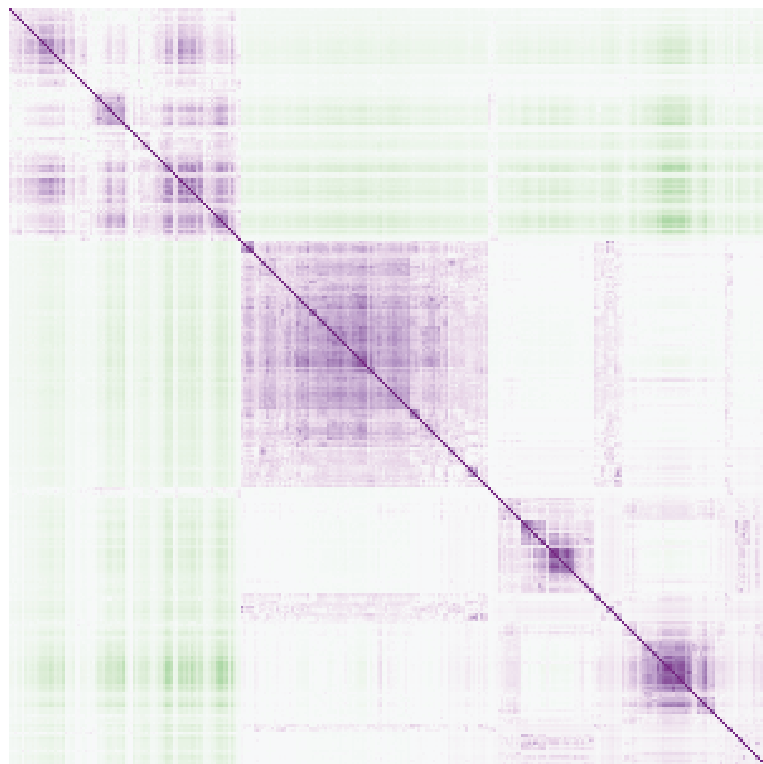

Transformed Correlations (C)

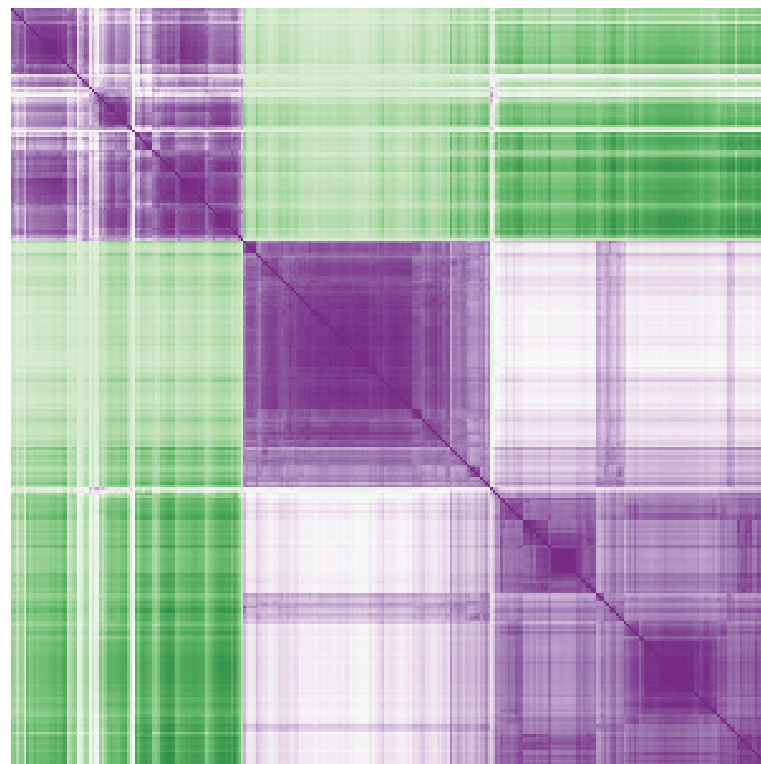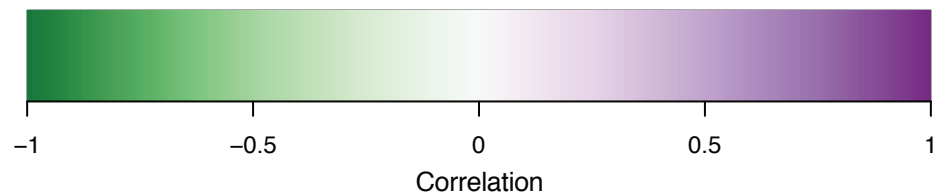

Supplement: Supplementary Figuer S1 [file ismej2016139x3.pdf]

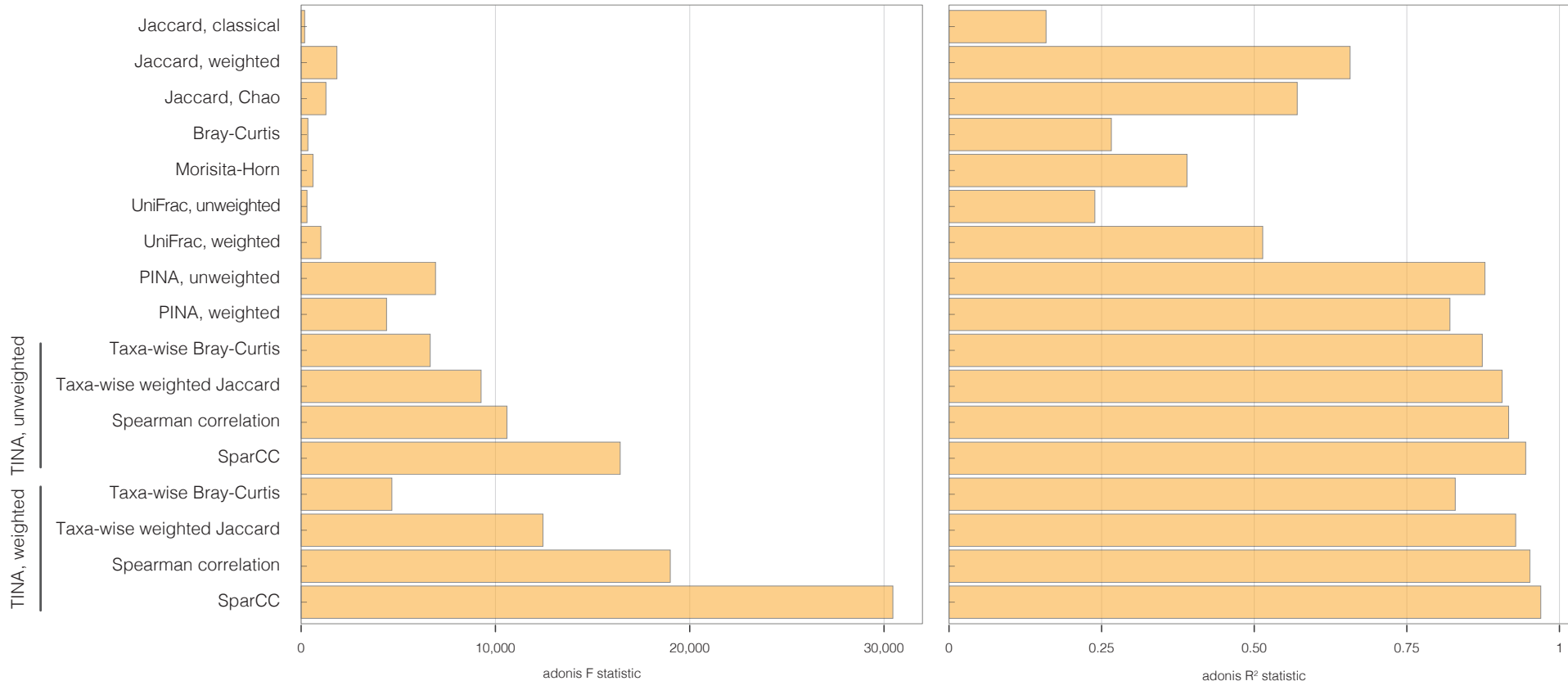

Supplement: Supplementary Figuer S2 [file ismej2016139x4.pdf]

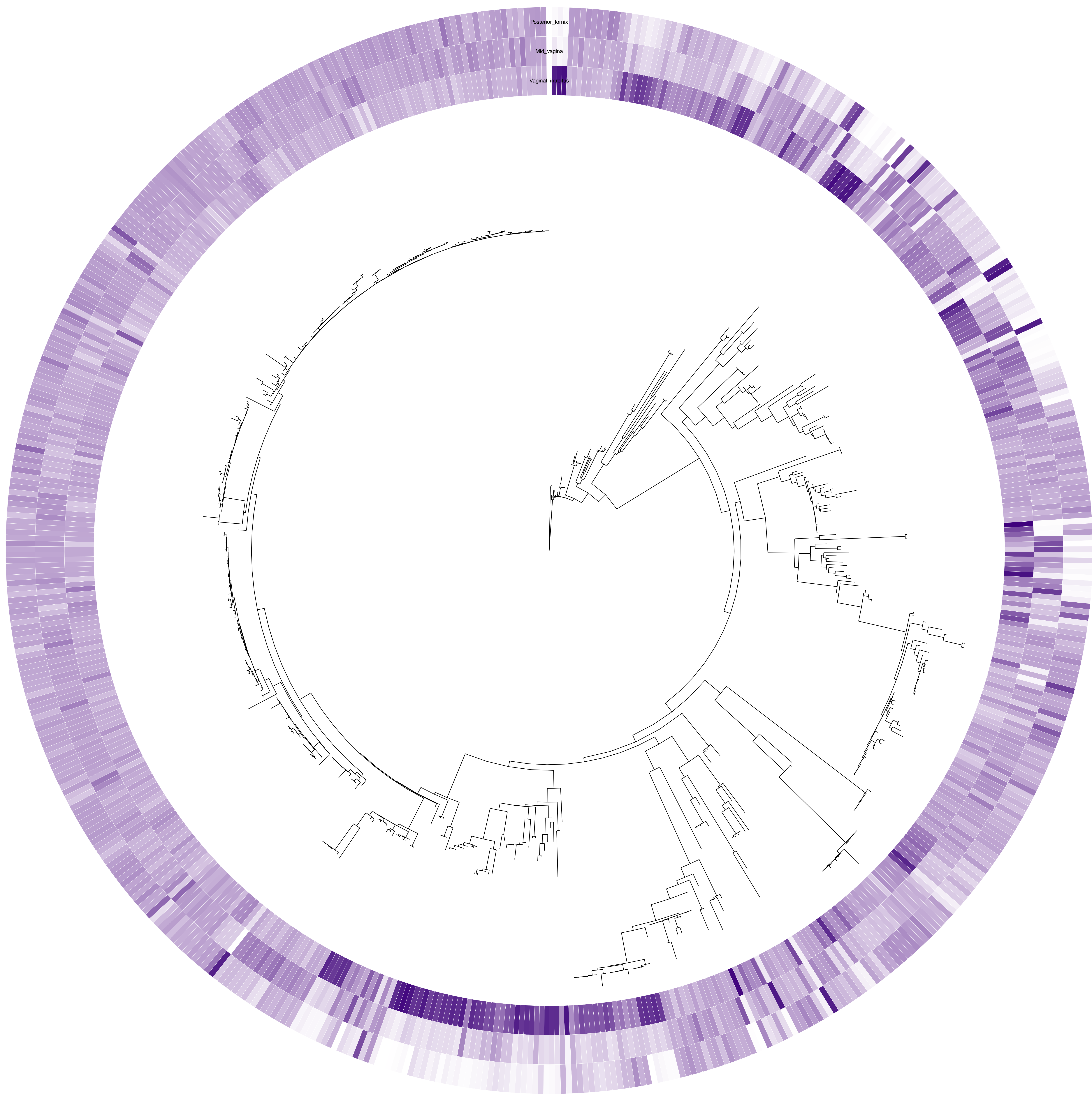

Supplement: Supplementary Figuer S3 [file ismej2016139x5.pdf]

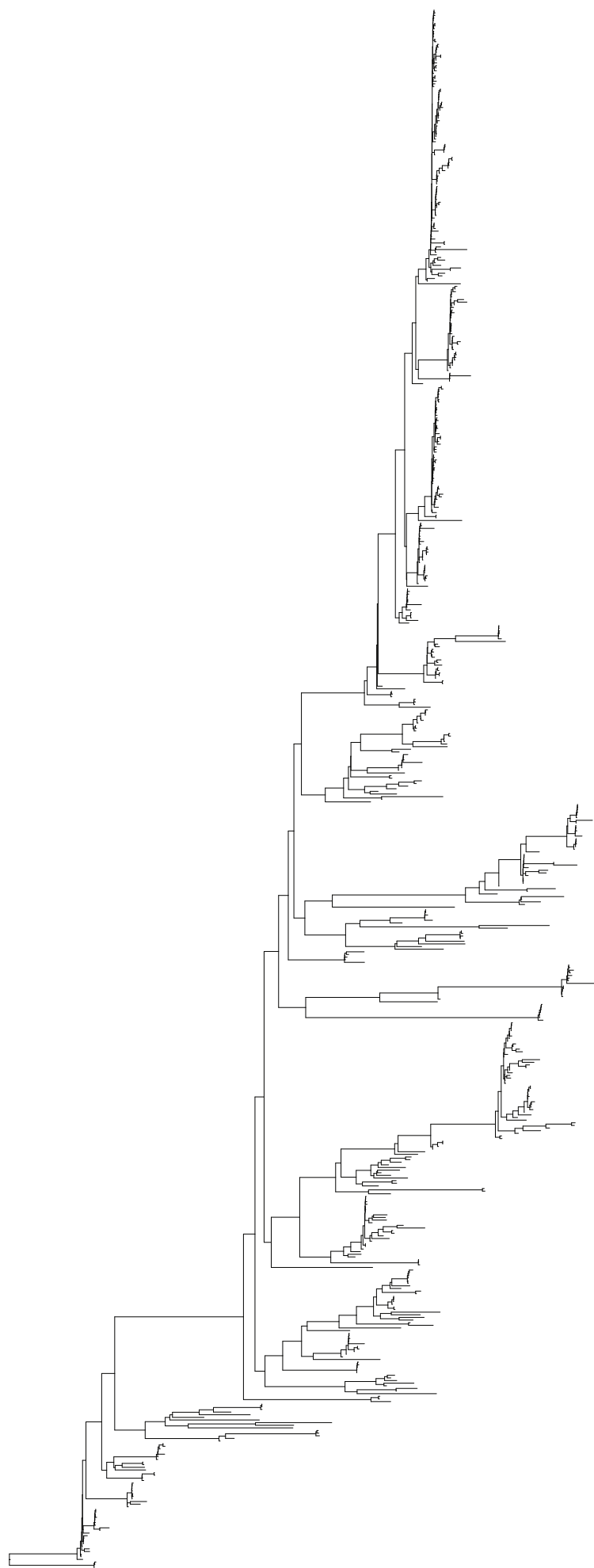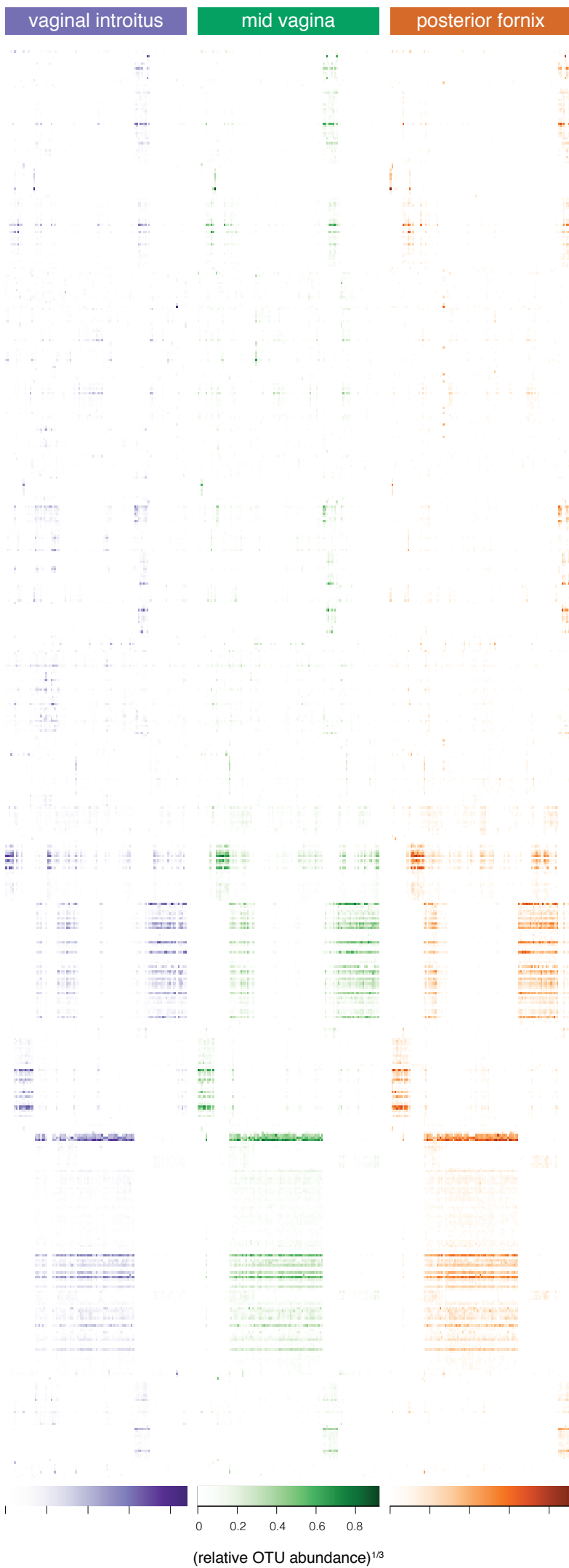

Supplement: Supplementary Figuer S4 [file ismej2016139x6.pdf]

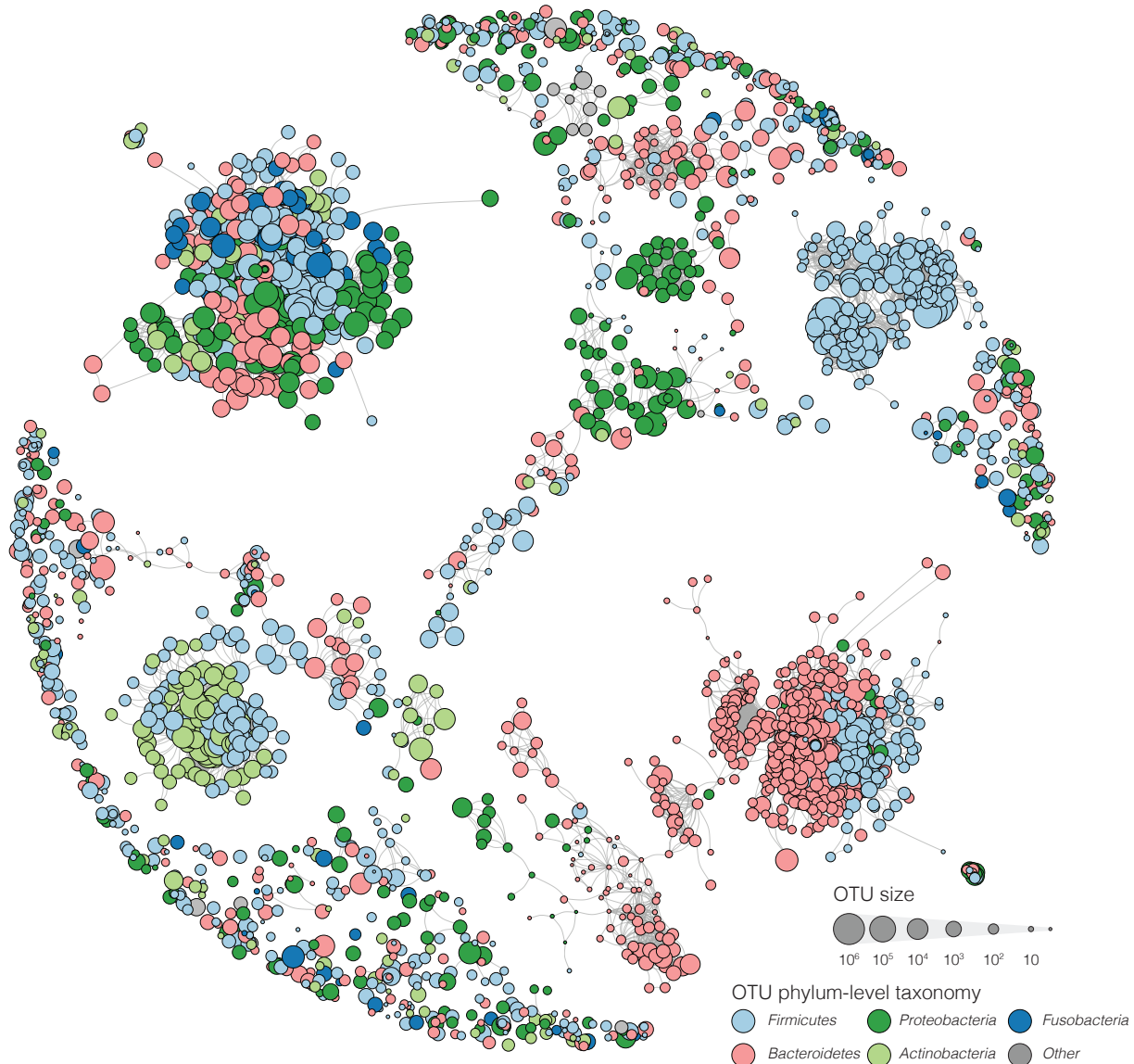

Supplement: Supplementary Figuer S5 [file ismej2016139x7.pdf]

A

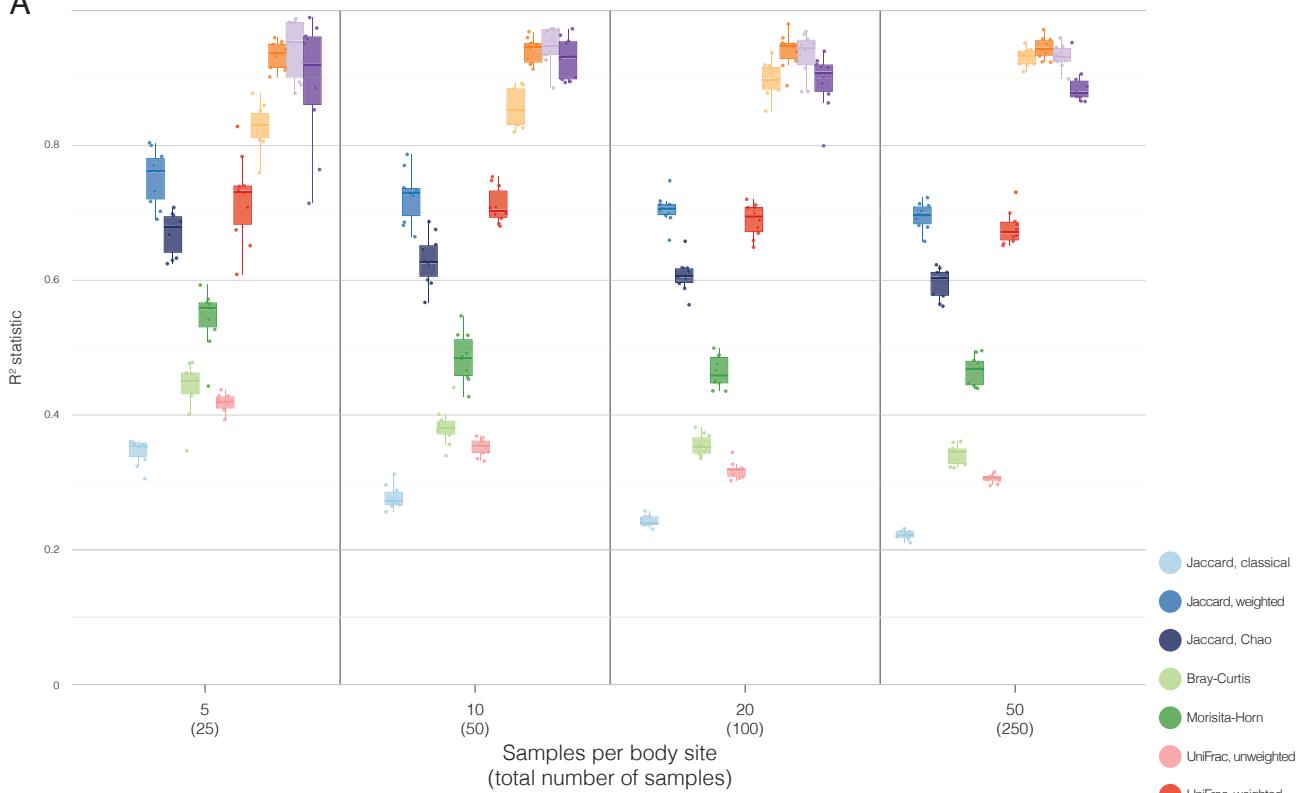

B

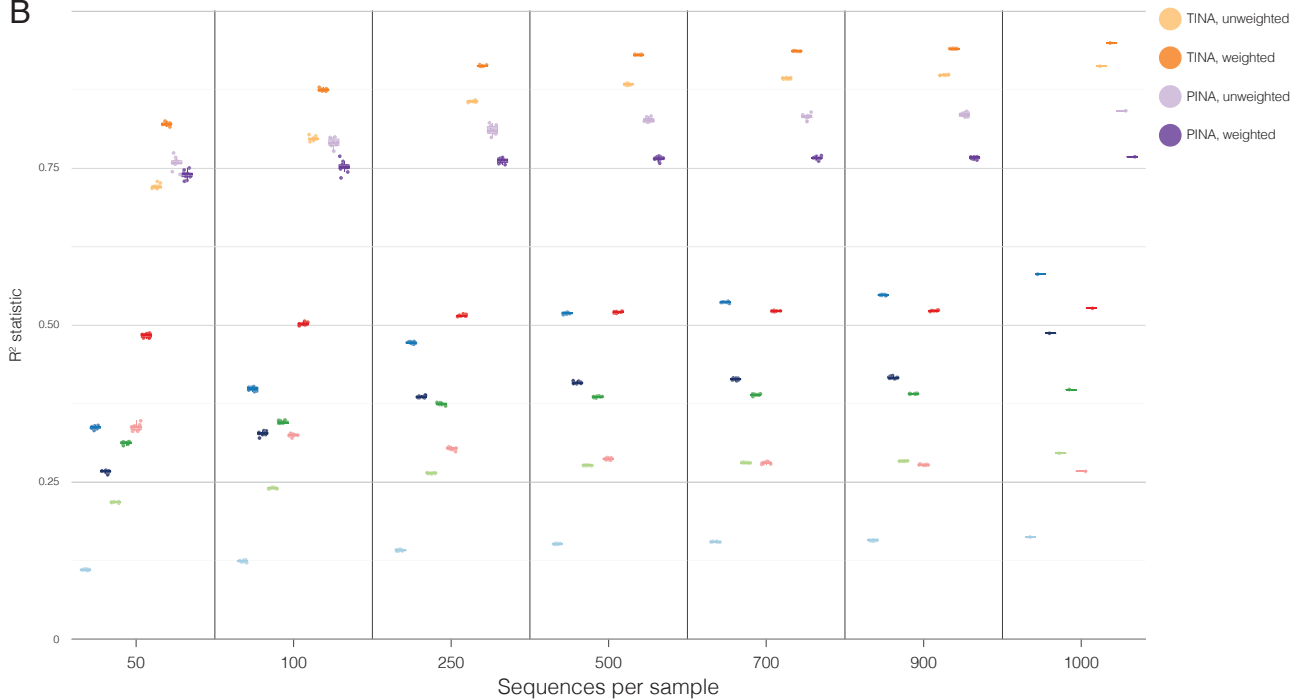

Supplement: Supplementary Figuer S6 [file ismej2016139x8.pdf]
