# Supplementary material for: A family of interaction-adjusted indices of community similarity
Source: ISME J. 2016 Dec 9;11(3):791–807. doi: 10.1038/ismej.2016.139 (PMC5322292; doi:10.1038/ismej.2016.139)
Supplement: Supplementary Information [file ismej2016139x9.doc]

Supporting Information

Table S1. Simulation results, raw adonis F and R2 values.

Table S2. Spearman correlations of community similarities to geographical distance for TARA samples.

Figure S1. Transformation of taxa co-occurrence into co-occurrence similarity smoothens and sharpens interaction information.

Heatmaps of correlation strengths are shown for 300 randomly selected OTUs from the set shown in the network of Figure 3, for SparCC co-occurrences (referred to as matrix I_C_ in the Methods section) and transformed SparCC correlations (matrix C). OTUs were clustered by SparCC correlation. Note that the color scale is [-1, 1] for a more intuitive visualisation, while we use a linear transformation to [0, 1] in our analyses.

Figure S2. Effect of co-occurrence network inference method on TINA performance.

PERMANOVA F and R^2^ values are shown for all tested methods (equivalent to Figure 2A), including TINA calculated based on four different taxa co-occurrence inference methods (taxa-wise Bray-Curtis, taxa-wise weighted Jaccard, Spearman correlation and SparCC; see main text).

Figure S3. A weak but significant phylogenetic signal for urogenital subsite.

A phylogeny of the most abundant OTUs in urogenital samples is shown with corresponding distributions of relative abundances (per OTU) across vaginal subsites. A weak but significant phylogenetic signal was detected for all three subsites(using the function multiPhylosignal from the R package picante; Kembel *et al.*, 2010).

Figure S4. Variation between subjects is larger than within subjects for urogenital samples.

The same tree as in Figure S3 is shown, but with OTU abundances across samples, clustered within urogenital subsites. While body-subsite specific variation in community composition was detected only using phylogenetic indices, variation between subject was significantly higher than within subjects for almost all metrics (see main text for further details).

Figure S5. HMP taxa co-occurrence network, annotated by taxonomy.

The same network as in Figure 3 of the main text, with nodes coloured by OTU phylum-level consensus taxonomy.

Figure S6. Down-sampling effects on body habitat partitioning, quantified as R^2^

This figure is analogous to Figure 5 of the main text, but showing down-sampling effects in terms of R^2^ instead of F statistics.
